# Spatio-temporal specialization of GABAergic septo-hippocampal neurons for rhythmic network activity

**DOI:** 10.1007/s00429-018-1626-0

**Published:** 2018-03-03

**Authors:** Gunes Unal, Michael G. Crump, Tim J. Viney, Tímea Éltes, Linda Katona, Thomas Klausberger, Peter Somogyi

**Affiliations:** 1 Department of Pharmacology, Mansfield Rd, University of Oxford, Oxford, OX1 3QT UK; 2 Department of Psychology, Bogazici University, 34342 Istanbul, Turkey; 3 Institute of Experimental Medicine, Hungarian Academy of Sciences, 1083 Budapest, Hungary; 4 Center for Brain Research, Medical University of Vienna, 1090 Vienna, Austria

**Keywords:** Basal forebrain, Septo-hippocampal, Hippocampus, GABAergic, Sharp wave-ripple, Theta

## Abstract

Medial septal GABAergic neurons of the basal forebrain innervate the hippocampus and related cortical areas, contributing to the coordination of network activity, such as theta oscillations and sharp wave-ripple events, via a preferential innervation of GABAergic interneurons. Individual medial septal neurons display diverse activity patterns, which may be related to their termination in different cortical areas and/or to the different types of innervated interneurons. To test these hypotheses, we extracellularly recorded and juxtacellularly labeled single medial septal neurons in anesthetized rats in vivo during hippocampal theta and ripple oscillations, traced their axons to distant cortical target areas, and analyzed their postsynaptic interneurons. Medial septal GABAergic neurons exhibiting different hippocampal theta phase preferences and/or sharp wave-ripple related activity terminated in restricted hippocampal regions, and selectively targeted a limited number of interneuron types, as established on the basis of molecular markers. We demonstrate the preferential innervation of bistratified cells in CA1 and of basket cells in CA3 by individual axons. One group of septal neurons was suppressed during sharp wave-ripples, maintained their firing rate across theta and non-theta network states and mainly fired along the descending phase of CA1 theta oscillations. In contrast, neurons that were active during sharp wave-ripples increased their firing significantly during “theta” compared to “non-theta” states, with most firing during the ascending phase of theta oscillations. These results demonstrate that specialized septal GABAergic neurons contribute to the coordination of network activity through parallel, target area- and cell type-selective projections to the hippocampus.

## Introduction

Projections from the medial septum (MS) of the basal forebrain to the hippocampal formation provide a key contribution to hippocampal network activity and resulting rhythmic oscillations (Petsche et al. 1962; Yoder and Pang 2005). These projections form part of a wider and diverse basal forebrain projection to the cerebral cortex (Detari and Vanderwolf 1987; Buzsaki et al. 1988; Gritti et al. 1997; Detari et al. 1999; Zaborszky et al. 1999, 2015; Duque et al. 2000; Manns et al. 2000a, 2003; Jones 2004; Duque and Zaborszky 2006; Kang et al. 2017). Selective inactivation of GABAergic medial septal neurons impairs spatial memory (Pang et al. 2011), similar to non-selective lesion or inactivation of the complete MS (Rawlins et al. 1979; McNaughton et al. 2006). GABAergic septal neurons exclusively innervate GABAergic interneurons in the hippocampus (Freund and Antal 1988; Unal et al. 2015), and the majority of their extra-hippocampal cortical postsynaptic neurons are also GABAergic cells (Freund and Buzsaki 1996; Gonzalez-Sulser et al. 2014; Unal et al. 2015). As septal GABAergic neurons often fire rhythmically, phase-coupled to hippocampal theta oscillations (Petsche et al. 1962), they have been proposed to be a key component of hippocampal theta genesis via disinhibition of principal cells (Freund and Antal 1988). Yet, the contribution of the different firing patterns of individual septo-hippocampal neurons to hippocampal network activity and to the coordination of principal cell assemblies remains to be explained.

Interactions between the medial septum and the hippocampus are reciprocal and dynamic depending on network states (Kocsis and Kaminski 2006; Hangya et al. 2009; Kang et al. 2017). Medial septal modulation of hippocampal principal cells via disinhibition by septal GABAergic afferents (Toth et al. 1997) is implemented as a rhythmic and sequential redistribution of inhibition over distinct subcellular domains of pyramidal cells through a variety of interneuron types (Somogyi et al. 2014). The temporal redistribution of inhibition depends on hippocampal network states, as different types of hippocampal interneuron show differential activity in relation to theta oscillations and sharp wave-ripple (SWR) events (Klausberger and Somogyi 2008). Many MS neurons show preferential firing at specific phases of the hippocampal theta cycle (King et al. 1998; Dragoi et al. 1999), and they may be active, remain silent, or may be inhibited during SWRs (Borhegyi et al. 2004; Viney et al. 2013). In the rat hippocampus, it is not known whether individual septal neurons that fire differentially during network oscillations uniformly innervate all types of hippocampal interneuron or are selective for particular types of interneurons. Specifically, it has been hypothesized that septal GABAergic neurons that discharge at the trough of hippocampal theta oscillations might innervate hippocampal interneurons terminating on the soma and proximal dendrites of pyramidal cells, whereas septal neurons that are activated at the peak of the hippocampal theta rhythm innervate those interneurons that terminate on distal dendrites of pyramidal cells (Borhegyi et al. 2004). In a recent testing of this hypothesis, Joshi et al. (2017) found that in the mouse hippocampus, a type of septo-hippocampal GABAergic neuron, the Teevra cell, was phase-coupled to the trough of CA1 theta cycles and selectively innervated theta peak firing axo-axonic neurons, but surprisingly preferentially in the CA3 area.

We have hypothesized that the GABAergic septo-hippocampal projection is composed of several types of selective and specialized neuron differentially involved in different brain states and tested this hypothesis in the rat. Establishing the spike-timing of GABAergic septo-hippocampal neurons during rhythmic hippocampal network events in combination with their specific hippocampal synaptic targets elucidates the mechanism underlying septal contribution to the hippocampal “chronocircuit” and the resulting rhythmic network activity.

## Materials and methods

### Experimental subjects and housing conditions

All procedures were performed under approved project and personal licenses at the University of Oxford in accordance with the UK Animals (Scientific Procedures) Act, 1986 and associated regulations under the approval of the UK Home Office and the Animal Care and Use Committees of the University of Oxford. Adult male Sprague–Dawley rats (*n* = 20; 250–350 g; Charles River) were housed in groups of two to four littermates per cage (19–22 °C; ~ 55% humidity; diurnal cycle, lights on from 8 am to 8 pm) with ad libitum access to food and water.

### Surgical procedures

Anesthesia was induced with 4% isoflurane (IsoFlo, Abbott) in medical oxygen (BOC Medical). It was maintained using an initial intraperitoneal injection of urethane (1.25 mg/kg; Sigma-Aldrich) followed by a 0.1 ml mixture of ketamine (20 mg/kg) and xylazine (2 mg/kg) administered after confirming that the animal had lost its eye blink reflex and failed to respond to a noxious foot pinch. Animals were mounted in a stereotaxic frame (Kopf Instruments) for the duration of the experiment. Craniotomy and duratomy were performed under analgesic treatment (subcutaneous injection of buprenorphine, Vetergesic; 0.03 mg/kg; and local injection of bupivacaine, Marcaine; 0.125% solution). After exposing and cleaning the skull, a craniotomy was performed 0.6 mm anterior of Bregma to expose the sagittal sinus. The craniotomy was widened, so that a glass electrode could later be targeted to the MS using a 15° latero-medial angle, 1.4 mm lateral to the central point of the sinus. A second craniotomy was performed 4.1 mm posterior and 2.2 mm lateral of Bregma to later target the dorsal CA1 with a glass electrode using a 10° postero-anterior angle. Duratomies were performed and sites were covered with sterile saline. Supplementary volumes of the same ketamine/xylazine mixture (0.01–0.05 ml; i.p.) were given throughout the experiment to maintain anesthesia and to induce slow wave (~ 1 Hz) activity (i.e., “theta”/“non-theta” periods; refer to definitions below). Every 2–3 h, a 2 ml glucose solution (5% in saline; Dechra) was administered subcutaneously. Six to twenty-four hours post juxtacellular labeling (see below), the animals were injected with an overdose of pentobarbital (20% wt/vol; i.p.) and perfusion fixed. Cardiac perfusion with saline was followed by 10–20 min fixation using 4% depolymerized paraformaldehyde (wt/vol, Sigma–Aldrich), 0.05% glutaraldehyde (wt/vol, distilled grade, TAAB Laboratories Equipment Ltd) and 15% (vol/vol) saturated picric acid (Sigma–Aldrich) in 0.1 M phosphate buffer (pH 7.4).

### In vivo extracellular single cell recording and juxtacellular labeling

Electrophysiological signals were simultaneously recorded from the MS and hippocampus. One filamented borosilicate glass pipette (1.2 mm outer diameter, 0.69 mm inner diameter, GC120F-10 Harvard Apparatus; tip diameter prepared to ~ 1.5 µm) was filled with 1.5%-3% neurobiotin (Vector Laboratories) in 0.5 M NaCl and inserted into the MS to record single cell activity and local field potentials (LFPs) via a chlorided silver wire within the glass pipette (17–30 MΩ). Another glass electrode was filled with 0–1.5% neurobiotin in 0.5 M NaCl and was lowered into stratum oriens of the dorsal CA1 to record LFPs. Electrophysiological signals were amplified 1000x (DPA-2FS and BF-48DGX amplifiers; npi electronic GmbH). Noise elimination was applied with HumBugs (Quest Scientific) for electrical (50 Hz) noise. The septal recording was split to form two channels. One channel was band-pass filtered at 0.8–5 kHz and digitized at 20 kHz (Power1401 A/D board, Cambridge Electronic Design) to reveal the action potentials. The other channel contained the LFPs and was band-pass filtered for 0.3–300 Hz at 1 kHz sampling rate. Acquisition of all signals was performed in parallel using Spike2 software (v7; Cambridge Electronic Design). After recording a neuron in the MS, the glass electrode was advanced into a “juxtacellular” position for labeling using neurobiotin (ELC-01MX amplifier, npi electronic GmbH or Neurodata IR 283A amplifier, Cygnus Technology). Briefly, once the spike amplitude was > 1 mV, positive current pulses (200 ms on, 200 ms off; ~1–10 nA) were delivered to the cell until spikes were entrained to the positive pulses (modulation) (Pinault, 1996; Duque and Zaborszky 2006). Neurons were modulated for 4–40 min to obtain long axonal labeling, with longer modulation times resulting in the most strongly labeled neurons. After the labeling attempt, the current pulses were ceased and the electrode was slowly moved away from the cell. The quality of the labeling depended on several factors, such as the exact position of the electrode tip in relation to the cell body, the duration and strength of the modulation achieved due to the stimulation and the type of recorded neuron.

### Juxtacellular labeling with recovery

In a separate experiment (animal M44), instead of urethane, anesthesia was maintained by continuous use of 1–3% isoflurane in medical oxygen to allow recovery of the animal for a longer post-labeling period. Following surgery as above, a single neuron in the MS was juxtacellularly labeled with 10% biotinylated dextran amines (BDA, 3 kDa) in 0.5 M potassium acetate (Sigma-Aldrich). Due to differences in brain state under isoflurane vs urethane, firing patterns were not analyzed. Antibiotic treatment was carried out post-operatively (enrofloxacin, Baytril; 0.1 ml; i.p.) and the animal was fixed by transcardial perfusion after 4 days.

### Analysis of network oscillations

Theta oscillations and SWR events were detected as reported before (Viney et al. 2013). Theta epochs were identified based on the theta (3–6 Hz) to delta (1–3 Hz) frequency power ratio being greater than 4 in three consecutive windows of at least 2 s. The beginning and end of detected theta periods were manually adjusted, if necessary. For detecting SWRs, the threshold was set to power values of 6× SD above the mean power in the ripple frequency band (90–200 Hz). The beginning and end of SWRs were identified when ripple oscillatory power exceeded or dropped below 2× SD above the mean power, respectively. Recordings were included in the analyses only if the number of detected theta cycles exceeded 44 and the number of detected SWRs was at least 12. The intervals outside detected theta epochs were defined as “non-theta” periods. “Inter-SWR” periods were derived from “non-theta” periods by excluding SWR times. Specifically, these include periods between two consecutive SWRs, between the end of a theta epoch and the beginning of a SWR, and between the end of a SWR and the beginning of a theta epoch. Thus, “non-theta” periods between two theta epochs that did not contain SWRs have not been considered.

Action potentials of septal neurons were sorted into 20 theta phase bins of 18° and 10 ripple phase bins of 36°, cycle-by-cycle. Each spike was assigned to an instantaneous theta and ripple phase, respectively, between two troughs (0° and 360°). For each neuron, we derived the theta and ripple phases of the recorded spikes. If these phases resulted in a non-uniform distribution around the theta and/or ripple cycle, we calculated the cell’s preferential mean phase of firing and the strength of its theta and/or ripple modulation, respectively (i.e., mean vector length) using Rayleigh’s method and normalized vector addition (Lasztóczi et al. 2011).

**Fig. 1:**
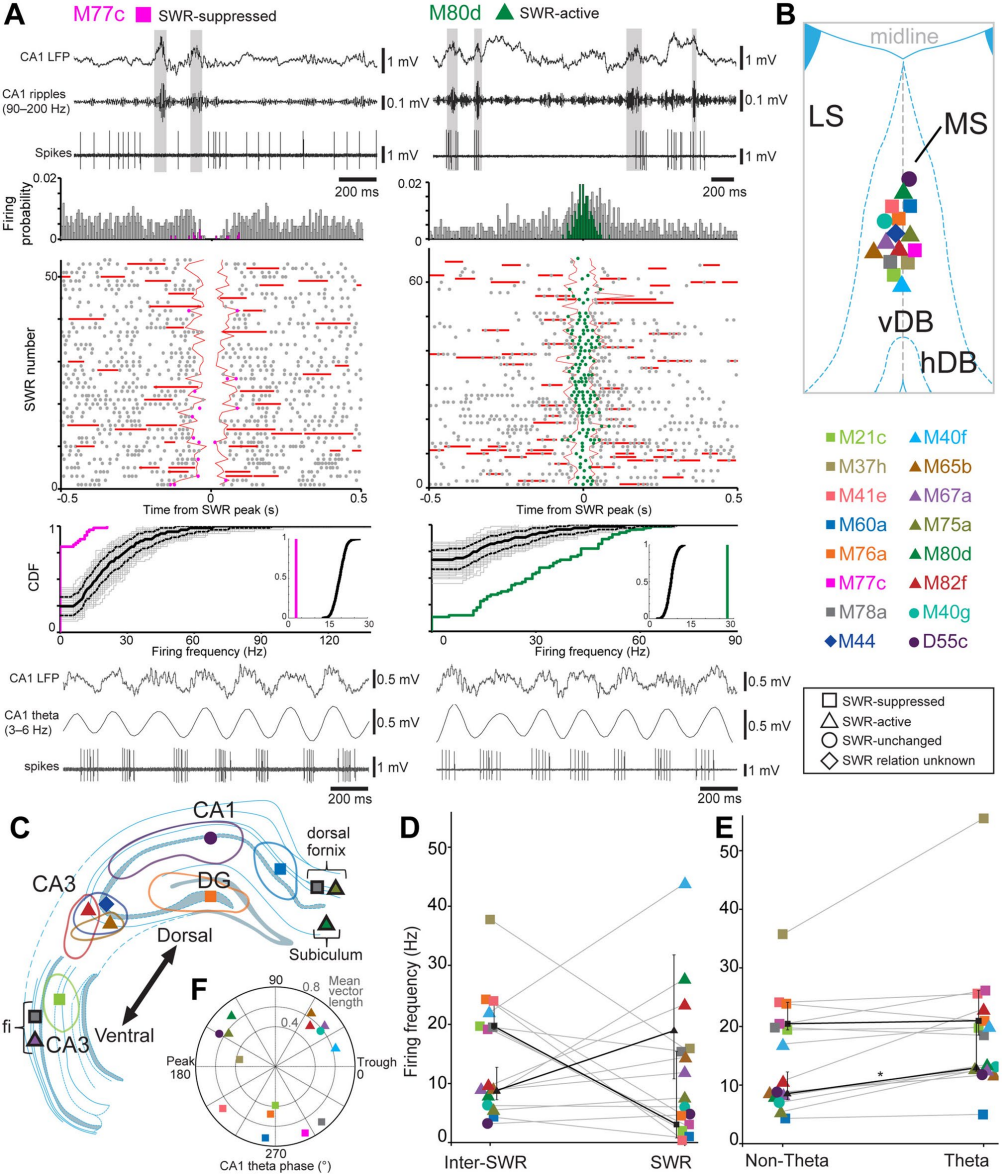
Comparison of labeled medial septal neurons by axonal target area, SWR-related activity, and theta phase preference. Labeled cells are color-coded for all panels. **a** Activity traces of SWR-suppressed neuron M77c (left) and SWR-active neuron M80d (right), which preferentially fire along the descending phase and ascending phase of theta cycles detected in stratum pyramidale of CA1 hippocampus (bottom left and right), respectively. Top left: the cell is inactive during SWRs (highlighted) during non-theta epochs. Middle left: average firing probability density and raster plot relative to all detected SWRs (*n* = 57). Firing probability during SWRs (magenta) and ± 0.5 s from the peak of SWR events (gray) and the cumulative distribution (CDF) of SWR rates show that M77c is a SWR-suppressed neuron. Top right: the cell fires during SWRs (highlighted) during non-theta epochs. Middle right: average firing probability density and raster plot relative to all detected SWRs (*n* = 67). Firing probability during SWRs (green) and ± 0.5 s from the peak of SWR events (gray) and the CDF of SWR rates show that M80b is a SWR-active neuron. Raster plots were aligned to the peak SWR power. SWR rate distributions were compared to surrogate SWR-rate distributions measured from outside SWR periods (see “Materials and methods”). **b** Approximate positions of labeled medial septal neurons. **c** Main hippocampal target areas (shown in the same hemisphere for convenience; dorsal CA3: M82f, M44; dorsal CA1: D55c, M60a; DG: M76a; dorsal subiculum: M80d; intermediate CA3: M21c). Black framed symbols represent the position of main axons, which could not be traced to terminations due to weak labeling (dorsal fornix: M75a, M78a; fimbria: M67a, M78a; dorsal subiculum: M80d). **d** Mean firing rates during SWR events compared to inter-SWR periods in non-theta epochs; neurons grouped (symbols) by analyzing firing rate distributions including all SWRs (see “Materials and methods”). Median (interquartile range, IQR) values of SWR-active (black triangles) and SWR-suppressed neurons (black squares) depict group-level changes. **e** Average firing rates outside (non-theta) and during CA1 theta epochs. SWR-active neurons (black triangles, median (IQR)) increase their firing rate during theta oscillations (asterisk, *n* = 6, Wilcoxon signed-rank test, *p* = 0.028); SWR-suppressed neurons show no group-level change (black squares, median (IQR), *n* = 7, *p* = 0.310). **f** Preferred mean firing phases of labeled neurons during theta oscillations recorded in the dorsal CA1 strata pyramidale/oriens. Labeled SWR-suppressed neurons (squares) fire sequentially along the descending slope of CA1 theta cycles. Most SWR-active neurons (triangles) fire along the ascending slope, but some also in the other quadrants. The radial axis shows the depth of theta modulation (mean vector length). *MS* medial septum, *LS* lateral septum, *vDB* vertical diagonal band, *hDB* horizontal diagonal band, *DG* the dentate gyrus, *fi* fimbria. Vertical scale bars, 0.5 mV; except for band-pass filtered CA1 ripples at the bottom of B, 0.1 mV

We analyzed the variability in firing rate of single neurons during hippocampal SWRs. As described previously (Katona et al. 2014), SWR-related firing rates have been compared to firing rates obtained outside SWR events. Firing rates have been calculated for the *n* detected SWRs. Next, a population of 1000× *n* “surrogate SWR” time windows were generated sequentially. “Surrogate SWRs” were restricted to “non-theta” periods, when the majority of SWRs occurred. For each of the 1000 sets, individual firing rates were calculated for the *n* “surrogate SWRs” and their average was derived. Due to the limited recording periods, each spike was included in “surrogate SWRs” repeatedly, but in different time frames. The firing rates during detected SWRs were compared to the average rates during “surrogate SWR” periods using a two-sample Kolmogorov–Smirnov (KS) test. Finally, for each neuron, a SWR index was calculated with values between − 1 (no firing during SWRs) and 1 (firing exclusively during SWRs), with 0 meaning no change in firing rate during SWRs compared to outside these events. Based on the KS test showing significant difference in firing rates, and using the SWR index we grouped septal neurons into “SWR-suppressed” (negative SWR index; e.g., M77c in Fig. 1a) and “SWR-active” (positive SWR index; e.g., M80d in Fig. 1a) populations. Some neurons were “SWR-unchanged”. This analysis reveals firing rate differences over the whole range of firing rates of the neuron during individual SWRs. The resulting categorization may result in a higher overall mean firing rate during all SWRs of a SWR-suppressed neuron (e.g., neuron M78a in Fig. 1) than the overall mean firing rate of a SWR-active cell during SWRs (e.g., neuron M67a in Fig. 1).

All analyses were carried out using Spike2 (v7, Cambridge Electronic Design) and MATLAB (v7.14-R2012a, MathWorks). To examine the variability and the reproducibility of our observations of different kinds of labeled MS neurons, we also analyzed long-duration recorded unlabeled neurons and included them in our statistical analyses. Unlabeled neurons were included in the dataset if recorded within ~ 500 µm of a labeled neuron that was confirmed to be in the MS (*n* = 13 unlabeled neurons from 7 rats) or had similar rhythmic firing patterns at a similar depth and brain state (*n* = 9 unlabeled neurons from 6 rats), as in other rats that had neurons previously labeled and identified.

### Immunohistochemistry and confocal imaging

Tissue preparation and anatomical analyses were carried out as described previously (Unal et al. 2015) using a wide-field epifluorescence microscope (Leitz DMRB; Leica Microsystems) equipped with PL Fluotar objectives and a laser scanning confocal microscope (Zeiss LSM 710; Zeiss Microscopy) equipped with DIC M27 Plan-Apochromat 40X/1.3 n.a., DIC M27 Plan-Apochromat 63X/1.4 n.a., and Plan-Apochromat 100X/1.46 n.a. oil immersion objectives. Membrane permeabilization was achieved using either tris buffered saline (TBS) containing 0.3% Triton X-100 detergent (TBS-Tx) or by performing two rounds of “freeze-thawing” (FT). Neurobiotin and BDA were visualized using streptavidin Alexa Fluor 488. Primary antibodies were detected by fluorophore-conjugated secondary antibodies (Unal et al. 2015). Initially, 1–4 primary antibodies were tested on each section. If required, additional primary antibodies were subsequently tested on the same sections. Two molecular markers expressed in different subcellular compartments were sometimes detected with the same fluorophore. The method specificity of immunohistochemistry was assessed by including parallel “negative control” brain sections that lacked the primary antibodies. Target molecule, host species and dilution for primary antibodies that we have reported previously in detail (Viney et al. 2013; Unal et al. 2015) are as follows: calbindin (CB), goat, 1:1000; CB, rabbit, 1:5000; cholecystokinin (CCK), guinea pig, 1:1000; CCK, rabbit, 1:500; choline acetyltransferase (ChAT), goat, 1:500; calretinin (CR), goat, 1:1000; CR, rabbit, 1:1000; GABA_A_R-α1, rabbit, 1:1000; gephyrin, mouse 1:500; hyperpolarization-activated cyclic nucleotide gated channel 4 (HCN4), mouse, 1:1000; Kv1.1, mouse 1:1000; N-terminal EF-hand calcium-binding protein 1 (NECAB1), mouse, 1:500; neuronal nitric oxide synthase (nNOS), rabbit, 1:1000; neuropeptide Y (NPY), rabbit, 1:5000; parvalbumin (PV), goat, 1:1000; PV, guinea pig, 1:5000; PV, rabbit, 1:500; PV, mouse, 1:5000; special AT-rich sequence-binding protein-1 (SATB1), goat, 1:400; SATB1, rabbit, 1:1000; somatostatin (SOM), mouse, 1:200; vesicular acetycholine transporter (VAChT), goat, 1:400; vesicular GABA transporter (VGAT), guinea pig, 1:500; VGAT, rabbit, 1:500; vesicular glutamate transporter 2 (VGluT2), guinea pig, 1:500. Information on the remaining primary antibodies are presented in Table 1.

**Table 1:** Information on host species, dilutions, sources, antigens and specificity of primary antibodies that have not been listed in Viney et al. (2013) or Unal et al. (2015)

Molecule	Host	Dilution	Source	Antigen	Specificity information
CB	Mouse	1:1000	Swant, code: 300	Purified calbindin from chicken gut	No specific labeling in knock-out mice (Airaksinen et al. 1997)
Gephyrin	Guinea pig	1:500	Synaptic Systems, code: 147004	Recombinant protein containing C-terminus amino acids 294–736	Labeling pattern as published with other antibodies
mGluR1a	Guinea pig	1:500	Dr. M. Watanabe, Hokkaido University, Japan	Rat protein amino acids 945–1127	Western blot (Nakamura et al. 2004)
	Goat	1:1000			Labeling pattern as published with other antibodies
VAChT	Rabbit	1:10,000	Sigma, code: V5387	Synthetic peptide C-terminus amino acids 512–530	Western blot (Rodriguez-Diaz et al. 2011)
VGluT2	Goat	1:500	Dr. M. Watanabe, Hokkaido University, Japan	C-terminus amino acids 519–582	Western blot (Miyazaki et al. 2003)
	Rabbit	1:500	Dr. M. Watanabe, Hokkaido University, Japan	Recombinant rat fusion protein amino acids 510–582	Western blot (Zhang et al. 2013)
VIP	Mouse	1:50,000	Dr. G. Ohning, UCLA, USA	Mouse peptide	Labeling pattern as published with other antibodies

### Analysis of the laminar distribution of axonal varicosities of a septo-CA3 neuron (M44)

In 24 coronal 70 µm-thick sections containing neuron M44, we have counted the total number of axonal varicosities (*n* = 1134) and determined their laminar distribution. To test if the varicosities were uniformly distributed across hippocampal layers, we have derived an expected uniform varicosity distribution and compared it to the measured distribution (Chi square test). First, we have quantified the volume fraction of each layer that contained axonal varicosities. In a two dimensional rendering of each section in Neurolucida (MBF Bioscience), two straight lines were drawn starting from the lateral end of the hippocampal fissure, and running through either the most medial or the most lateral varicosities of the axon in CA3. These two lines were then connected at their ends, producing a trapezoid or a scalene triangle, if their origin already converged on the hippocampal fissure. Next, the laminar boundaries were drawn which intersected the lines demarcating the medial and lateral boundaries of the axon. The laminar areas measured in each section were multiplied by 70 µm, the average section thickness, and summed up to extrapolate the innervated volume of each hippocampal layer. Then, we have generated 1000 surrogate sets of the 1134 varicosities distributed within the total volume of all layers innervated by the axon and, for each set, we have extracted the expected number of varicosities per layer under the assumption of uniform distribution using the layer sub volume ratios calculated above. The expected uniform varicosity distribution was derived as the median of the 1000 surrogate sets.

### Analysis of synaptic target selectivity of septo-hippocampal theta-coupled neuron (D55c) by immunohistochemical characterization of postsynaptic elements

Boutons of septo-hippocampal neuron D55c were tested for possible apposition to soma and dendrites immunoreactive for at least one of 10 tested molecules. Some of these, such as NPY and SOM, are mainly present in somata and much less in dendrites. Others, such as nNOS or PV, are present in both somata and dendrites. Depending on the interneuron type, some of the molecules can be co-expressed by some, but not all neurons. Boutons were most frequently tested for three non-overlapping cell group markers: PV, CR and nNOS. Following immunoreaction, the tests were carried out by analyzing Z-stacks of high resolution confocal microscopic images (see above for details). A putative synaptic apposition was assumed when no gap could be identified between the neurobiotin-labeled septal bouton and the putative postsynaptic hippocampal soma or dendrite in any dimension of a complete confocal Z-stack. Because this MS neuron was a GABAergic neuron and most GABAergic synapses contain the synapse-specific postsynaptic protein gephyrin, a subset of appositions were tested for gephyrin immunoreactivity (*n* = 94) and they were all immunopositive. This confirmed that the microscopic prediction alone provided high confidence for synaptic junctions.

To test if boutons from the septo-hippocampal neuron contacted PV or CR or nNOS-positive profiles with the probability of their availability as postsynaptic structures originating from neurons expressing these molecules, we assessed the uniformity of target distributions. Our null hypothesis was that if the distribution of boutons was uniform, they would then contact interneuron somata and dendrites in proportion to their relative surface areas. The surface areas of most interneuron types in CA1 are not known; hence, we could not account for all synaptic targets, but restricted our surface estimates to PV, CR and nNOS-expressing neurons. First, we have calculated an estimate of the average surface area of PV+ and CR+ neurons using published data (Gulyas et al. 1999) in all hippocampal layers except stratum lacunosum-moleculare, which was not innervated by this septo-hippocampal neuron.

Next, for estimating the average surface area of nNOS+ Ivy cells, we used reconstructions of 3 nNOS+ Ivy cells (neuron T134a; Fuentealba et al. 2008; neuron D26p; Lapray et al. 2012; neuron PL310812; Lau et al. 2017) and derived their surface in the layers innervated by the septo-hippocampal neuron (T134a, soma surface: 352.4 µm^2^, dendritic surface: 5686.0 µm^2^; D26p, soma surface: 536.5 µm^2^, dendritic surface: 10920.3 µm^2^; PL310812, soma surface: 467.9 µm^2^, dendritic surface: 6966.0 µm^2^). Dimensions were calculated using shrinkage factors measured as applied to our processing conditions, and presented as values expected in the perfusion fixed brain, which undergoes shrinkage from the live dimensions. One Ivy cell soma was close to the border of stratum radiatum and stratum lacunosum-moleculare (Lau et al. 2017). Some of its dendrites were partially lost due to errors in tissue processing. The lengths of missing parts of the dendrites were extrapolated based on dendrites with natural ends within the well-processed sections. Namely, we assumed that the cut branches of dendritic trees would have the same average surface area as the other branches of their branch order. Each incomplete dendritic branch was pruned back to the closest branching point where a virtual branch with the same total downstream surface as the average branch of that order was placed. Starting from the highest order of incomplete dendritic branch, the downstream surface areas of all complete branches of that order were calculated. For example, if the highest order of an incomplete branch was 6th order, we used other 6th order branches that formed natural endings and calculated their downstream surface area. Some of these dendritic branches had 7th or 8th order sub-branches, whose surface areas were also added to the surface of the appropriate 6th order root branch. Once there was a total downstream surface area for each 6th order branch, these were then averaged to get an estimate for the missing branches. All incomplete branches of the same order had identical total downstream surface areas allocated by this method. For lower order branches, the estimates of the total downstream surfaces included the newly calculated estimated surfaces of incomplete higher order branches.

For comparison of the innervation of PV+ and CR+ targets, we first established the proportions of boutons/postsynaptic profiles that were tested for both for PV and CR (*n* = 42). The three categories were PV+ only (*n* = 22, 52%), CR+ only (*n* = 2, 5%) and PV−/CR− (*n* = 18, 43%). We used these proportions to predict the distribution of these three categories among targets to boutons that were tested only for the expression of either PV (*n* = 88) or CR (*n* = 16). Of the 88 putative target profiles tested only for PV, 49 were observed as PV+. The remaining 39 PV-negative targets were proportioned into predicted CR+ only (*n* = 4, 10%) and PV−/CR− (*n* = 35, 90%) postsynaptic profiles, as derived from the targets tested for both molecules above. Similarly, in the target sample tested for only CR, the 16 CR− targets (100%) were proportioned as putative PV+ only (*n* = 9, 55%) and PV−/CR− (*n* = 7, 45%). To estimate the target distribution of the 146 boutons, we took the sum of the population tested for both PV and CR, and the derived populations tested only either for PV or CR. This resulted in 80 predicted PV+ targets, 6 predicted CR+ targets, and 60 predicted PV−/CR− targets. Then, the distribution of the 86 targets, each positive for either PV or CR (derived from measured and estimated numbers), was compared to the expected distribution of targets based on the proportion and cell surface ratios of PV+ and CR+ neurons. The ratio of PV+ basket cells (BCs), axo-axonic cells (AACs) and bistratified cells to CR+ interneuron-specific interneurons I and III was previously estimated as 1.675:1 across all hippocampal layers by (Bezaire and Soltesz 2013). Interneuron-specific interneurons II (CR+) were left out, since the overwhelming majority of their dendrites are located in stratum lacunosum-moleculare (Acsády et al. 1996), which was not innervated by this septo-hippocampal neuron.

The same procedure was repeated for predicting the expected proportions of PV and nNOS-positive targets. For this, 43 boutons were tested for both PV and nNOS, and targets were either only PV+ (*n* = 16, 37%) or both nNOS− and PV− (*n* = 27, 63%). No nNOS+ profile was in apparent contact with these boutons. An additional 16 boutons were tested only for nNOS and all targets were immunonegative. In this latter population, the expected number of PV+ targets was 6 (37% of the 16 targets). Among the additional 88 boutons tested only for PV, 49 were observed as PV+. Summing the above targets detected or calculated as positive for either PV (71) or nNOS (0) resulted in 71 targets. Based on a 1.054:1 ratio of nNOS+ Ivy cells to PV+ interneurons (Fuentealba et al. 2008) in all layers of the hippocampus except stratum lacunosum-moleculare, and the relative surface areas, we derived an expected uniform distribution of the 71 putative targets as 49 PV+ and 22 nNOS+.

For the comparison between all three target interneuron groups, the observed and derived samples were summed. This resulted in 86 targets, of which n = 80 were PV+, *n* = 6 were CR+ and none were nNOS+ profiles. Based on the relative numbers and surface ratio of PV+, CR+ and nNOS+ cells, a uniform distribution of boutons would give 55 PV+, 7 CR+ and 24 nNOS+ targets. These two distributions were compared statistically (Chi square test) to test for any target specificity of the axon of septo-hippocampal neuron D55c.

### Statistical analyses

For each septal cell, the depth of its theta and/or ripple modulation was determined using the Rayleigh test and the preferential mean theta and/or ripple phase of its firing was computed using normalized vector addition. We calculated the mean depth of theta modulation and the mean preferential theta phase of firing (circular mean ± circular standard deviation) of defined groups of septal neuron using circular statistics. We compared these parameters between the groups by permutation tests (Good 2000). Mean firing rates were compared between theta epochs and periods without theta oscillations (see the definition above) using the Wilcoxon signed-rank test. Mean firing rates during SWR episodes were compared with those during “surrogate SWR” periods using two-sample Kolmogorov–Smirnov (KS) tests (see above). Uniformity of targeted postsynaptic profile distribution was determined using Chi square tests. Analyzed targets were immunopositive for one of the two or three tested (i.e., PV, CR and nNOS) and mutually exclusive molecular markers. For all statistical analyses, the test statistic and the p value are reported at the significance level *α* = 0.05. All tests were carried out in SPSS (v23, IBM) and MATLAB (Statistics Toolbox, MathWorks).

### Visualization of axon terminals and neuron reconstruction

Resin-embedded, osmium-treated, serial coronal cut brain sections of 60–80 µm thickness reacted for horseradish peroxidase (HRP) using 3,3′-diaminobenzidine (DAB) as chromogen and H_2_O_2_ as substrate were used for light microscopic visualization of fine axonal processes including axon terminals, as previously described (Tukker et al. 2013). Digital reconstructions in 3D were performed using Neurolucida (MBF Bioscience). A Nikon Eclipse 80i transmitted light microscope equipped with a VC Plan Apo 100x/1.4 NA oil immersion objective and a Lucivid display (MBF Bioscience) was used in continuous mode. Correction against tissue shrinkage has been applied as described previously (Tukker et al. 2013). In brief, sections were expanded in the *X* and *Y* dimensions on average by 14.4% following TBS-Tx, or 4% after “freeze-thawing” treatment. The thickness of each section was measured and expanded to the cut section thickness of 60, 70 or 80 µm. Consecutive sections were aligned to match segments of axons and dendrites. Further shrinkage correction in *X* and *Y* dimensions was applied to obtain the best alignment, if needed. For some illustrations, parts of neurons were manually traced (Fig. 5g, h) using a drawing tube attached to a transmitted light microscope equipped with a Plan-Apochromat 63x/1.4 n.a. oil immersion objective.

## Results

Extracellular recordings and juxtacellular labeling of MS neurons under anesthesia (*n* = 38 recorded cells, and attempted labeling) resulted in 16 labeled neurons (Fig. 1b) and 11 axonal arborizations outside the MS (Fig. 1c; Table 2). Immunohistochemical testing (Table 3) showed VGAT in axon terminals (8/8 tested neurons), PV (*n* = 15/16 tested neurons) in somata and/or proximal dendrites, as also shown by retrograde axonal labeling (Unal et al. 2015), nuclear immunoreactivity for SATB1 (*n* = 14/14 tested neurons), immunoreactivity for the hyperpolarization-activated cyclic nucleotide gated channel 4 (HCN4; *n* = 12/12 tested neurons) and voltage-gated potassium channel Kv1.1 (*n* = 6/10 tested neurons) along the somato-dendritic membrane (Varga et al. 2008). Tested neurons lacked detectable immunoreactivity for calcium-binding proteins NECAB1 (*n* = 12), CR (*n* = 10) and CB (*n* = 2). The dendrites in the MS were predominantly non-spiny (*n* = 13/15 cells with labeled dendrites; *n* = 2/15 with sparse spines towards their distal ends; Table 2). The main axons originated either from somata (*n* = 8) or proximal dendrites (*n* = 3; Table 2). Notably, 9 out of 12 visualized axons had an initial hooked profile, heading first ventrally for 40–300 µm and then turning dorso-caudal. Axon collaterals were identified for 8 out of 11 neurons within the MS, including two neurons innervating the triangular septal nucleus.

**Table 2:** Anatomical characteristics of labeled medial septal neurons

Neuron	Dendrite	Axon origin	Hooked axon	Local septal axonal varicosities	Route of main axon
D55c	Non-spiny	Soma	No	++	Fimbria
M40g	Non-spiny	Soma	Yes	–	nt
M21c	Spiny	Soma	No	+	Fimbria
M37h	Non-spiny	Soma	No	nt	nt
M41e	nt	nt	nt	nt	nt
M60a	Spiny	Soma	Yes	++	Dorsal fornix
M76a	Non-spiny	Soma	Yes	+	Fimbria
M77c	Non-spiny	nt	nt	+	nt
M78a	Non-spiny	Dendrite	Yes	–	Dorsal fornix and fimbria
M40f	Non-spiny	nt	nt	nt	nt
M65b	Non-spiny	nt	nt	–	Fimbria
M67a	Non-spiny	Soma	Yes	–	Fimbria
M75a	Non-spiny	nt	Yes	–	Dorsal fornix
M80d	Non-spiny	Dendrite	Yes	+++	Dorsal fornix
M82f	Non-spiny	Soma	Yes	++++	Fimbria
M44	Non-spiny	Dendrite	Yes	+	fimbria

*nt*, could not be tested or observed due to insufficient labeling or other technical problems; ‘+’ signs denote relative density of observed axonal varicosities; – signifies lack of any local axonal varicosity in ≥ 6 consecutive ~ 70 µm-thick coronal medial septal sections

**Table 3:** Immunohistochemical molecular markers or marker combinations found in different groups of labeled medial septal neurons

Neuron	Immunohistochemical test									
	PV	CB	CR	NECAB1	SATB1	HCN4	Kv1.1	VGAT	VGluT2	VAChT
SWR-unchanged neurons										
D55c	+, s, d	nt	nt	nt	+, s	+, d	–, s, d	+, a	–, a	–, a
M40g	+, s, d	nt	–, d	–, d	nt	nt	nt	nt	nt	nt
SWR-suppressed neurons										
M21c	–, s, d, a	–, s, d	–, s, d	–, d	+, s	+, s, d	nt	+, a	nt	nt
M37h	+, s, d	nt	–, d	–, d	+, s	+, d	+, d	nt	nt	nt
M41e	+, s, d	nt	nt	–, s, d	+, s	nt	–, s, d	nt	nt	nt
M60a	+, a	nt	–, d	nt	+, s	+, d	+, s, d	+, a	–, a	nt
M76a	+, d	nt	nt	–, d	+, s	+, s, d	nt	+, a	nt	nt
M77c	+, s, d	nt	–, d	–, d	+, s	nt	+, s, d	+, a	nt	nt
M78a	+, s, d	nt	–, d	–, s, d	+, s	+, s, d	+, s, d	nt	nt	nt
SWR-active neurons										
M40f	+, s, d	nt	–, s, d	nt	+, s	nt	nt	nt	nt	nt
M65b	+, s, d	nt	–, d	–, d	+, s	+, s, d	+, d	nt	nt	nt
M67a	+, s, d	nt	nt	nt	+, s	+, s, d	nt	nt	nt	nt
M75a	+, s, d	nt	nt	–, d	+, s	+, s, d	–, s, d	nt	nt	nt
M80d	+, d	–, s, d	–, d	–, d	+, s	+, s, d	+, s, d	+, a	nt	nt
M82f	+, s, d	nt	–, d	–, d	+, s	+, s, d	–, d	+, a	nt	nt
SWR relation unknown										
M44	+, d, a	nt	nt	–, d	nt	+, d	nt	+, a	–, a	–, a

+, immunopositive; –, no immunoreactivity detected in the labeled neuron while other immunopositive cells were identified nearby; *nt*, not tested; *s, d, a*, tested on the soma, dendrite, axon, respectively

The overall mean firing rates ranged from 4.4 to 43.6 Hz (mean ± SD = 17.36 ± 10.5, *n* = 37 cells recorded under urethane, ketamine and xylazine anesthesia; see Methods). These firing rates are within the ranges reported in earlier studies on identified neurons in the basal forebrain using urethane anesthesia, for GAD-positive cells ~ 18 Hz (Manns et al. 2000a), for PV-positive cells ~ 40 Hz (Duque et al. 2000; Varga et al. 2008), for HCN positive cells 13–19 Hz (Varga et al. 2008), for cholinergic cells 5–14 Hz (Manns et al. 2000b; Duque et al. 2000), for putative cholinergic and other cell types (Detari and Vandewolf 1987; Detari et al. 1999; Borhegyi et al. 2004) or in freely moving rats (Buzsaki et al. 1988). Changes in the level of anesthesia resulted in differences in the amount of different oscillatory network states, which we defined as “theta” (periods with prominent 3–6 Hz oscillations) and “non-theta” (periods lacking theta oscillations; Fig. 1).

### Differential firing of medial septal neurons during hippocampal sharp wave-ripples

Hippocampal SWRs reflect highly coordinated increased discharge of neurons lasting ~ 100 ms. We have detected SWRs in CA1 (90–200 Hz; *n* = 12–116 per recording; Table 4; Fig. 1a) during non-theta network states. The firing rate of most individual MS neurons (Fig. 1d) during SWRs was significantly different to that during periods outside SWRs (*p* < 0.05 for *n* = 31/37 neurons, KS tests for firing rate distributions during SWRs versus “surrogate SWR” periods, “Materials and methods”; including 9 labeled neurons; Table 4). A subset of neurons did not significantly change their firing rate during SWRs compared to outside SWRs (*p* > 0.37 for *n* = 6/37 cells, KS tests; including 2 labeled neurons; Fig. 1d). From the neurons that changed their firing rates during SWRs, we found that 14/37 increased their firing during SWRs (SWR index > 0.10; Table 4; Fig. 1a, d; includes 6 labeled neurons) and 17/37 decreased their firing (SWR index < 0.10; Table 4; Fig. 1a, d; includes 7 labeled neurons). Data from 12 neurons in the SWR-active group reported previously (Viney et al. 2013) are included for comparison. The action potentials of 3 out of 14 analyzed SWR-active neurons (40 ± 15 SWRs per cell) were coupled to hippocampal ripple oscillatory cycles (mean angles 216.5° for M65b, 134.5° for M75a and 106.5° for M79a; Rayleigh Z test, p values 0.03, 0.08 and 0.08, respectively). Of these neurons, M65b and M75a had projection axons that could not be traced to their terminations. A representative SWR-suppressed neuron M77c (Fig. 1a, left) had a mean SWR firing rate of 3.03 Hz (SWR index: -0.73; “surrogate SWRs”, 19.18 Hz) and was strongly theta modulated. In contrast, a SWR-active neuron M80d (Fig. 1a, right) had a mean SWR firing rate of 27.73 Hz (SWR index: 0.56; “surrogate SWRs”, 7.84 Hz) and was also strongly theta modulated.

**Table 4:** Physiological activity patterns of all recorded medial septal neurons during hippocampal theta oscillations and SWR events

Neuron	Mean theta phase of firing (deg.)	Mean theta vector length of firing (r)*	Mean firing rate, non-theta freq. (Hz)	Mean firing rate, theta freq. (Hz)	Mean number of spikes per theta cycle (SD)	Theta cycle count	Mean firing rate during SWRs (Hz)	Mean firing rate, of surrogate SWRs (Hz)	SWR index**	(p), SWR firing rate comparison: Kolmogorov–Smirnov test	SWR count
SWR-unchanged neurons											
**D55c**	150.80	0.63850	8.67	11.70	2.8 (0.7)	715	4.70	3.19	0.19	0.627586	68
M40b	294.20	0.61700	37.34	37.00	9.5 (2.3)	1140	34.97	37.41	− 0.03	0.434968	53
**M40g**	38.80	0.59031	7.11	13.10	3.7 (2.1)	213	6.03	6.26	− 0.02	0.371590	49
M66b	275.99	0.33157	8.12	8.40	3 (1.8)	2129	8.38	8.08	0.02	0.909448	20
M80b	112.69	0.49001	10.59	23.80	5.9 (3.8)	1691	7.82	8.25	− 0.03	0.986206	94
M82b	283.23	0.66308	15.89	9.10	2.4 (1)	1294	14.14	16.76	− 0.08	0.955708	35
SWR-suppressed neurons											
**M21c**	270.56	0.39783	19.40	19.80	4.9 (1.6)	881	1.91	19.68	− 0.82	0.000016	14
M22c	288.50	0.81819	16.96	17.42	3.9 (1.1)	355	3.90	16.91	− 0.63	0.002220	18
M30e	305.60	0.36712	18.22	19.40	4.7 (1.7)	2361	3.79	19.03	− 0.67	< 0.00001	25
M31g	267.38	0.61670	9.22	11.69	3.3 (2.1)	78	0.00	9.36	− 1.00	0.010166	15
M37b	96.80	0.56896	8.51	13.70	4.1 (1.4)	124	1.12	9.06	− 0.78	0.042998	20
M37e	64.61	0.70234	10.93	14.80	4.7 (2.1)	463	2.62	10.92	− 0.61	0.042038	44
M37f	330.31	0.52629	11.86	11.00	3.4 (2.1)	325	2.27	11.56	− 0.67	0.000080	30
**M37h**	171.96	0.37500	35.69	55.50	15.3 (7.2)	497	15.87	37.79	− 0.41	0.000044	57
**M41e**	220.73	0.67442	24.12	25.60	7.1 (3.6)	1046	0.24	23.94	− 0.98	< 0.00001	52
**M60a**	262.50	0.72908	4.35	5.00	1.7 (0.9)	628	0.79	4.35	− 0.69	0.035504	43
M74b	224.48	0.68309	33.09	20.70	5.3 (1.4)	1163	21.70	34.21	− 0.22	0.000668	17
**M76a**	264.82	0.49227	23.86	20.90	5 (2.8)	2547	4.48	24.13	− 0.69	< 0.00001	35
M77b	267.22	0.17907	15.92	42.50	10 (2.3)	386	2.01	15.88	− 0.77	< 0.00001	116
**M77c**	295.96	0.73012	20.36	26.10	5.6 (1.8)	5368	3.03	19.18	− 0.73	< 0.00001	57
**M78a**	310.00	0.72527	19.83	18.50	4.4 (2.1)	706	15.39	19.57	− 0.12	0.036443	71
M82a	327.48	0.58180	12.49	7.94	2.3 (1.2)	1524	1.22	13.22	− 0.83	< 0.00001	35
M82e	178.90	0.21709	43.33	44.80	11.3 (5.5)	128	3.78	46.02	− 0.85	< 0.00001	21
SWR-active neurons											
M31b	38.55	0.43392	10.05	11.40	3.3 (2.2)	1443	34.07	8.29	0.61	< 0.00001	46
M31h	303.53	0.52617	41.72	46.09	12.4 (5)	306	67.72	40.59	0.25	< 0.00001	46
M40d	33.20	0.51999	4.63	8.60	2.2 (1.1)	425	11.79	3.98	0.50	0.004131	12
**M40f**	16.20	0.63768	16.98	20.00	5.2 (3.2)	789	43.89	22.06	0.33	< 0.00001	57
M41d	70.96	0.46451	16.58	31.80	7.9 (4.2)	44	30.56	14.93	0.34	0.010170	22
M60d	207.28	0.64577	9.61	15.07	3.8 (1.1)	578	13.74	9.62	0.18	0.011395	47
**M65b**	56.19	0.66666	8.84	11.70	2.7 (1.3)	1543	14.44	8.81	0.24	0.000333	30
**M67a**	39.39	0.64298	8.43	12.60	3.2 (1.3)	489	11.86	8.84	0.15	0.002674	25
M74c	318.30	0.75708	14.27	28.90	7.3 (4.1)	1241	30.56	12.65	0.41	0.000029	42
**M75a**	144.06	0.58351	5.45	12.80	3.5 (0.9)	578	7.57	5.48	0.16	0.005440	41
M77a	269.93	0.73417	16.25	49.90	10 (3.6)	3252	39.62	16.31	0.42	0.000055	41
M79a	14.78	0.68242	10.35	24.05	5.4 (2)	1547	17.39	8.19	0.36	< 0.00001	57
**M80d**	129.93	0.67793	8.11	13.40	3.5 (1.2)	71	27.73	7.84	0.56	< 0.00001	67
**M82f**	49.82	0.54672	10.69	23.05	6 (3.1)	919	23.42	9.64	0.42	< 0.00001	27

Bold, juxtacellularly labeled neurons

*Values of r between 0 and 1, maximally correlated = 1

**See “Materials and methods”

All neurons were significantly coupled to theta oscillations (*n* = 37/37 tested cells), some of which also increased their firing rate during theta oscillatory periods (Fig. 1e). Most SWR-suppressed neurons (*n* = 14/17) fired preferentially along the descending slope of CA1 theta cycles (mean angle 271.5°, range 64.6–330.3°, Rayleigh Z test, *p* = 0.0092), whereas most SWR-active septal neurons (*n* = 10/14) preferentially fired along the ascending slope (range 14.8–144.1°). However, we encountered neurons within both groups that were in different quadrants (Fig. 1f; Table 4). The SWR-unchanged group (Table 4) also showed a range of theta phase preferences (287.34° ± 74.83°, *n* = 6). There was no difference in the strength of theta coupling between SWR-suppressed (mean *r* = 0.55 ± 0.18) and SWR-active neurons (mean *r* = 0.61 ± 0.1; *p* = 0.65, permutation test, difference = 0.0622, Fig. 1f). The firing frequency of SWR-suppressed (*Z* = − 1.396, *p* = 0.163, *n* = 17) group did not differ during non-theta and theta oscillatory periods. However, SWR-active neurons significantly increased their firing during CA1 theta oscillations (22.1 ± 13.02 Hz) compared to non-theta (13 ± 9.15 Hz) periods (*Z* = − 3.296, *p* = 0.001, *n* = 14, Wilcoxon signed-rank test). This difference between SWR-suppressed and -active groups is also evident for the labeled neurons (Fig. 1e, medians in black). It should be noted that the mean firing rate of SWR-active neurons outside theta periods (13 ± 9.15 Hz) is lower than the mean non-theta firing rate of SWR-suppressed neurons (19.30 ± 10.35 Hz), whereas the firing rate of both groups is similar during theta oscillations (SWR-active: 22.1 ± 13.02 Hz vs SWR-suppressed: 22.08 Hz ± 13.63). Finally, the mean number of spikes per theta cycle was not different between SWR-suppressed and -active neurons (5.7 ± 3.5 vs 5.5 ± 3; mean ± SD spikes, *n* = 17 and 14 neurons, respectively; *U* = 114, *p* = 0.843, Mann–Whitney *U* test). In summary, the firing patterns revealed three groups of strongly theta rhythmic neurons: SWR-suppressed neurons with no change in mean firing rate across network states, SWR-active neurons that increased their firing from non-theta to theta network states, and a group that did not significantly change firing during SWRs.

### Hippocampal synaptic target selectivity of a theta-coupled medial septal neuron

**Fig. 2:**
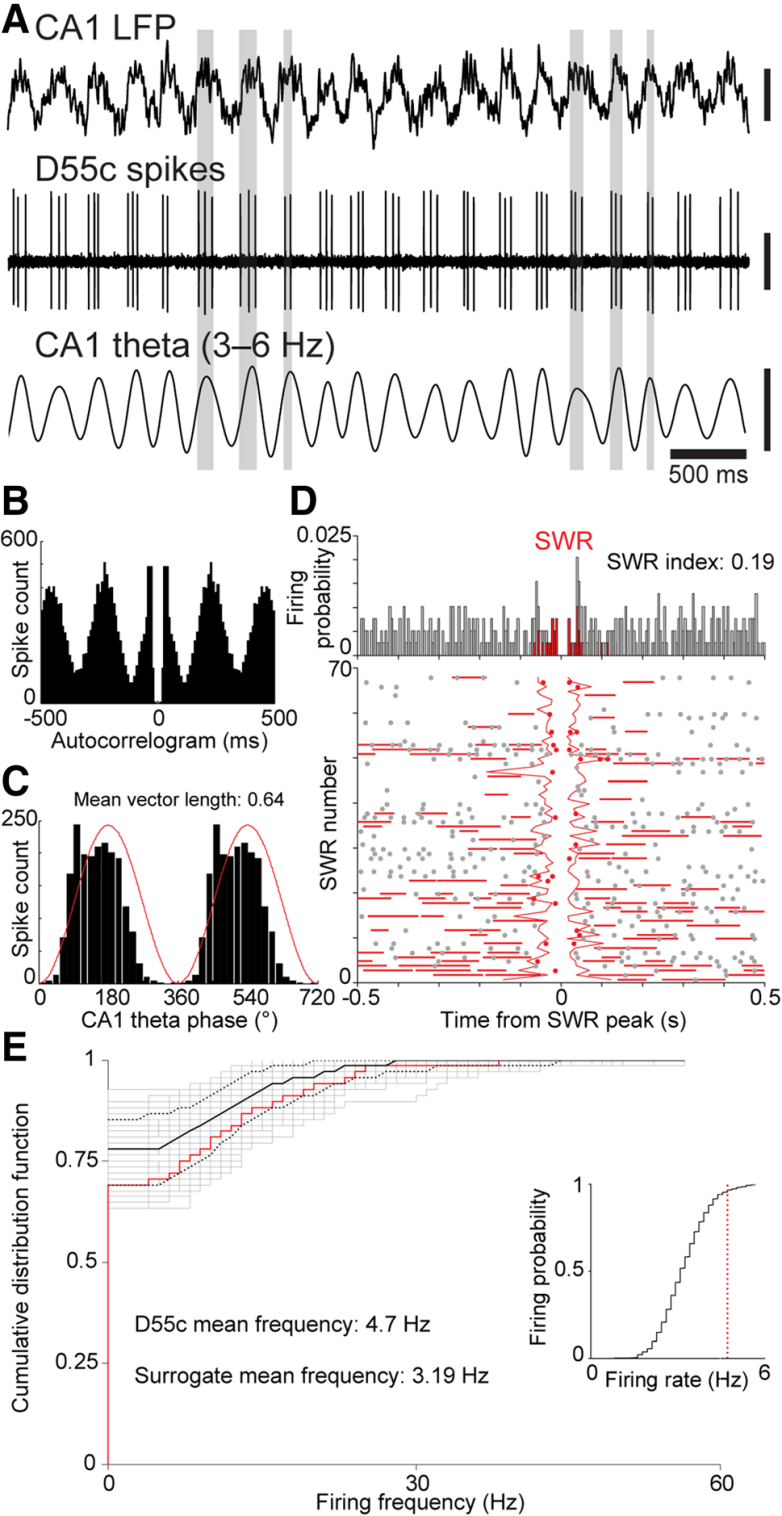
Firing patterns of GABAergic medial septal neuron D55c projecting to the dorsal CA1 and subiculum. **a** Recorded traces of the cell’s activity in relation to theta oscillations in the CA1 stratum pyramidale (LFP, top; band-pass filtered LFP is at the bottom). The neuron shows preferential firing at the late ascending phase/peak of the theta cycles (some highlighted). **b** Autocorrelogram of the neuron’s firing during theta epochs showing rhythmicity at theta frequency. **c** Theta phase histogram from all theta epochs (data duplicated digitally; red line, sinus function). **d** Firing probability histogram (top) and raster plot (bottom) of firing relative to SWRs (*n* = 68) during non-theta epochs. **e** Left, Comparison of mean firing rates of D55c during SWRs and periods outside SWR events. The firing rates during individual SWRs (red), displayed as a cumulative distribution function (CDF), are mostly within the distribution of a surrogate set of 1,000 firing rate-distribution during the same periods outside SWR events (gray; median, solid black line; 95% confidence intervals, broken lines). On average, there is no change in firing rate during SWRs. Right, comparison of mean SWR-related firing rate (red) with the distribution of surrogate mean SWR-related rates (black). Vertical scale bars, 0.5 mV

Neuron D55c was immunoreactive for PV, SATB1, HCN4 and VGAT (Table 3) and preferentially fired on the late ascending slope of theta cycles (150.8 ± 54.43; Fig. 2a–c). The neuron did not change firing rate during SWRs (*n* = 68) if the whole duration of SWRs was measured (SWRs, 4.7 ± 8.04 Hz, surrogate SWRs, 3.19 ± 0.8 Hz; *p* = 0.6276, KS test; SWR index: 0.19; Fig. 2d, e), and was, therefore, grouped as a SWR-unchanged neuron. However, the neuron was silent at the peak of ripple power (Fig. 2d); the spikes within the standard definition of SWR periods occurred at the beginning and at the end.

**Fig. 3:**
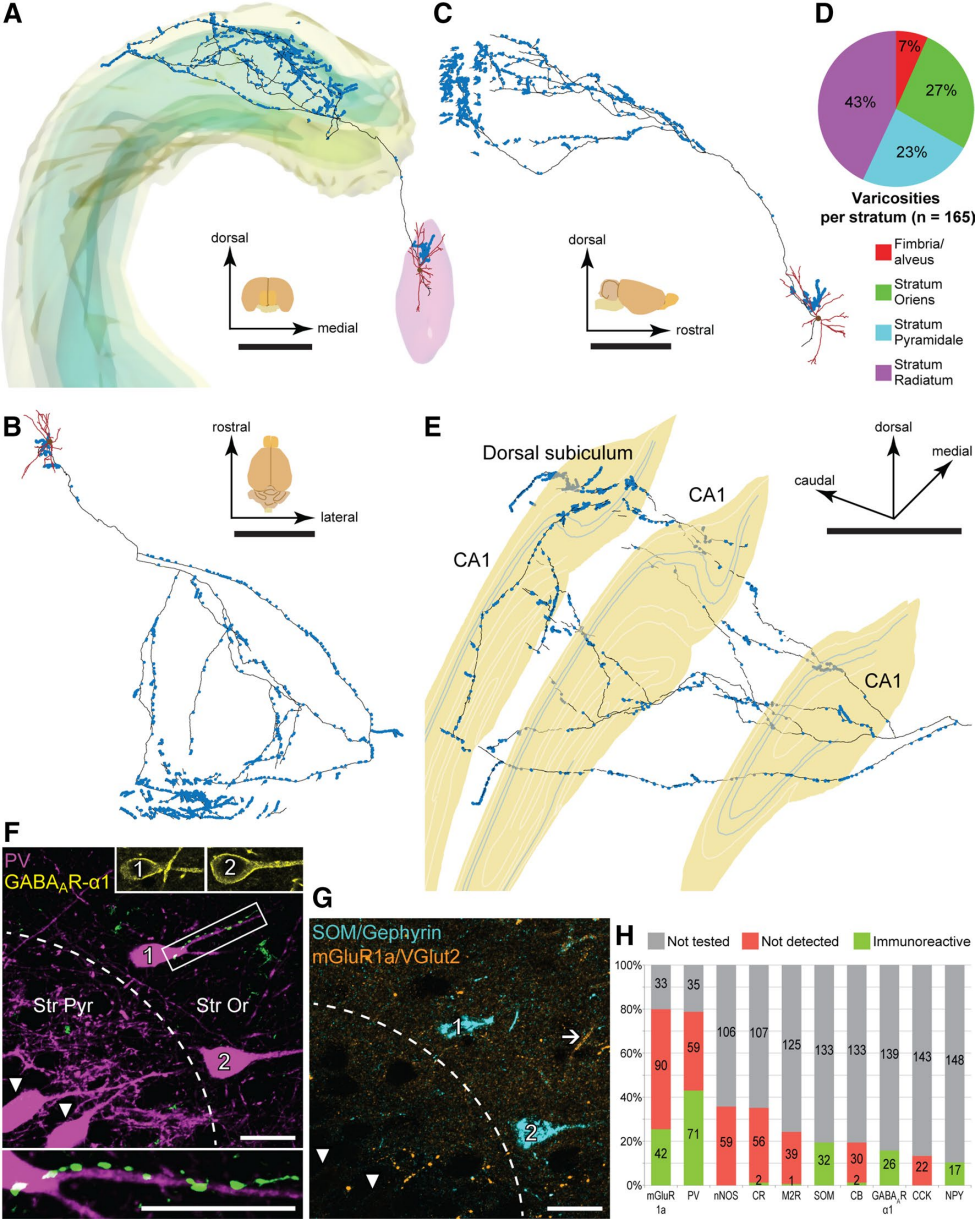
Projection patterns and targets of theta-coupled GABAergic medial septal neuron D55c. **a–c** Digital reconstruction of the neuron in frontal (**a**), top (**b**) and side (**c**) views showing the soma and dendrites (brown) in the MS (pink, **a**) and the axon (black) bearing varicosities (blue). Parts of the axon lost in 2 sub-optimally processed sections appear as gaps. The frontal view includes the contours of the pyramidal and granular layers (green) in the hippocampus (yellow, **a**). **d** Laminar distribution of axonal varicosities in CA1 tested for postsynaptic molecular cell markers. **e** Reconstruction of the axon after its first branching point at an oblique angle shows innervation of the CA1 and dorsal subiculum. Three single sections (yellow) are represented along the axon. Blue contours mark borders of stratum pyramidale. **f, g** Two bistratified cells (1, 2) in stratum oriens of the CA1 are immunoreactive for PV (**f**), GABA_A_R-α1 (**f**, upper insets) and SOM (**g**), but lack detectable immunoreactivity for mGluR1a (**g**; arrow, an immunopositive profile). The soma and a proximal dendrite of bistratified cell 1 are in apposition to septal boutons (green; framed area in **f**, enlarged in the inset below). Two other PV-immunopositive, SOM/mGluR1a-negative neurons in stratum pyramidale (arrowheads, **f, g**) were not observed to be contacted by the septal axon. **h** Normalized distribution of immunoreactivity for each tested molecule in presumed postsynaptic targets of septal boutons (*n* = 165, numbers within columns). Many axonal targets were tested for several molecules in sequential reactions. Individual targets were contacted by 1–16 boutons. **f, g** Maximum intensity projections of confocal image stacks; 2.93 µm-thick. Median filter was applied (*x, y, z*: radius 1 pixel) in **f, g**. Scale bars, 1 mm in **a**–**e**, 20 µm in **f, g**. Scale bar in F applies to the small insets

The axon emitted local collaterals in the MS (*n* = 314 axonal varicosities; Fig. 3a–c) then innervated the most septal part of the hippocampus with few terminals, continuing to dorsal CA1 (*n* = 656) and adjoining subiculum (*n* = 449), and forming numerous en passant and terminal varicosities. In CA1, 43% of varicosities were in stratum radiatum, 7% in the fimbria/alveus, 27% in stratum oriens, and 23% in stratum pyramidale (Fig. 3d, e). Labeled axonal varicosities were GABAergic synaptic boutons evidenced by gephyrin-immunopositive postsynaptic puncta (*n* = 94/94 tested varicosities). Thus, this septo-hippocampal neuron simultaneously provides rhythmic GABAergic input to restricted parts of the MS, CA1 and the subiculum.

Most varicosities were in apposition to dendrites (Fig. 3f–h), which we tested for 1–5 of ten different molecular markers in different combinations (Fig. 3h; Table 5), and some targeted somata (*n* = 5 tested). Of the total of 165 axonal boutons tested, 105 (64%) were in close apposition to immunopositive postsynaptic profiles. Because not all boutons were tested for all molecules, the postsynaptic structure with unknown identity to 60 boutons either did not contain the molecule that was tested for, or the molecule it contained was not tested. Of all the postsynaptic profiles tested for PV (*n* = 130), 71 (55%) were i mmunopositive (Table 5). Synaptic targets were tested for metabotropic glutamate receptor 1a (mGluR1a; *n* = 124 tested) and some were immunopositive (*n* = 42, 34%); 11 of these (26 tested) were positive for PV as well. Molecules, like NPY and SOM, are mainly located in somata and less in dendrites (Fig. 3H); thus, immunonegative dendrites tested for these molecules may represent false negative targets.

**Table 5:**
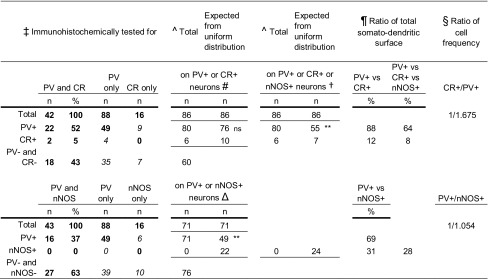
Postsynaptic interneurons, tested for PV, CR and nNOS, to 165 boutons of D55c in CA1

+, immunopositive; −, immunonegative; *ns*, non-significant

^^^Sum of measured data and extrapolated from measured, calculated, plain font

^#^Tested + extrapolated vs expected *χ*2 (1, *n* = 86) = 1.811; *p* = 0.178

^Δ^Tested + extrapolated expected (**) *χ*2 (1, *n* = 71) = 31.878; *p* < 0.001

^†^Tested + extrapolated vs expected (**) *χ*2 (2, *n* = 86) = 28.707, *p* < 0.001

^¶^Calculated from relative cell frequencies and somato-dendritic surface ratios, plain font

^§^Bezaire and Soltesz, 2013; Fuentealba et al. 2008, plain font

^‡^Bold, measured data; italics, extrapolated from measured

We tested potential synaptic target selectivity by evaluating immunoreactivity for non-overlapping molecular cell type markers PV, CR and nNOS (Jinno and Kosaka 2002; Bezaire and Soltesz 2013). We imaged 165 boutons (130 for PV, 58 for CR, 59 for nNOS) with putative postsynaptic profiles, and tested some for several molecules (Fig. 3H; Table 5). Based on targets tested for both PV and CR (*n* = 42 boutons), and for both PV and nNOS (*n* = 43) in addition to extrapolated numbers from tests for one molecule only, we calculated that a total of 86 boutons targeted 80 PV+, 6 CR+ and 0 nNOS+ postsynaptic elements. We assessed whether the frequency of PV+, CR+ or nNOS+ targets were different from what would be expected if this septo-hippocampal GABAergic neuron contacted these interneurons uniformly according to their frequency in CA1. First, the relative numbers and surface fraction of these cells were calculated in the innervated layers (Table 5). This required the mean surface area of nNOS-positive ivy cells, which we estimated from new calculations (“Materials and methods”) as 8309 ± 2813 µm^2^ (*n* = 3) Taking into account the relative proportions of these cell types (Fuentealba et al. 2008; Bezaire and Soltesz 2013), the cell surface ratios of PV+ and CR+ cells (Gulyas et al. 1999) and that of our measurement of nNOS+ ivy cells, we derived the relative proportions of cell surfaces of PV:CR:nNOS cells as 7.86:1.00:3.43 in the innervated layers (“Materials and methods”). A uniform innervation of these surfaces gives 55 PV+, 7 CR+ and 24 nNOS+ neuronal profiles which was significantly different from the detected distribution (Table 5).

To identify the source of this difference, we first calculated the distribution of PV+ and CR+ targets (estimated, 80 PV+ and 6 CR+, out of 86), based on the observed numbers (71/130 for PV, 2/58 for CR) including the profiles tested for both PV and CR (“Materials and methods”). After taking into account the frequency and the surface area of PV+ and CR+ cells, their relative contributions were 33,009 µm^2^ (PV, 88%) and 4,388 µm^2^ (CR, 12%) respectively. We estimated that a uniform distribution should result in 76 PV+ and 10 CR+ postsynaptic profiles (“Materials and methods”). The above distributions are not significantly different (Table 5), suggesting that the PV+ and CR+ target interneurons are innervated uniformly in the CA1. Next, we compared the detected and expected frequency of PV+ and nNOS+ targets. After accounting for the relative frequency of nNOS+ and PV+ interneurons and differences in their estimated surface area, we have derived the proportions of PV (19,714 µm^2^, 69%) to nNOS (8,758 µm^2^, 31%) cell surfaces. A uniform axonal innervation pattern would give 49 PV+ and 22 nNOS+ target profiles of the 71 boutons in the calculation, which is significantly different from the derived distribution of recorded targets (71 PV+ vs. 0 nNOS+; Table 5). This shows that D55c selectively innervates PV+ targets over nNOS+ profiles.

We have identified 4 postsynaptic interneurons, within or close to the pyramidal cell layer, as bistratified cells based on positive immunoreactivity for PV and SOM. Target interneurons were also immunopositive for mGluR1a (2 cells) and both NPY and GABA_A_R-α1 (2 cells, Fig. 3f–g) as reported previously (Baude et al. 2007). Eleven varicosities were in close apposition to a bistratified neuron (3 on soma; 8 on a dendrite, Fig. 3f), another bistratified neuron was contacted by 16 boutons (5 on soma; 11 on dendrites) and a third by 6 boutons (3 on soma; 3 on dendrites). In the fourth case, a single bouton was on a proximal dendrite. In total, four postsynaptic PV and SOM-immunoreactive bistratified neurons received 32 axonal varicosities (31%, soma; 69% dendrites).

Bistratified, AAC, O-LM, one kind of BC and a few projection neurons express PV in rat CA1 (Klausberger and Somogyi 2008). Bistratified cells expressing SOM and NPY (Klausberger et al. 2004) represent 24% of PV+ cells in and close to stratum pyramidale (Baude et al. 2007). If D55c innervated PV+ cell types uniformly, we would expect two or three BCs, one or no AAC and a single bistratified cell out of four target PV+ neurons. The probability of detecting four target bistratified cells sequentially is low (*p* = 0.25^4^ = 0.0039), indicating that this septo-hippocampal neuron selectively targets bistratified cells over other PV-immunopositive cell types such as BCs and AACs.

### A theta-coupled medial septal neuron targeting PV+ interneurons in the dentate gyrus

**Fig. 4:**
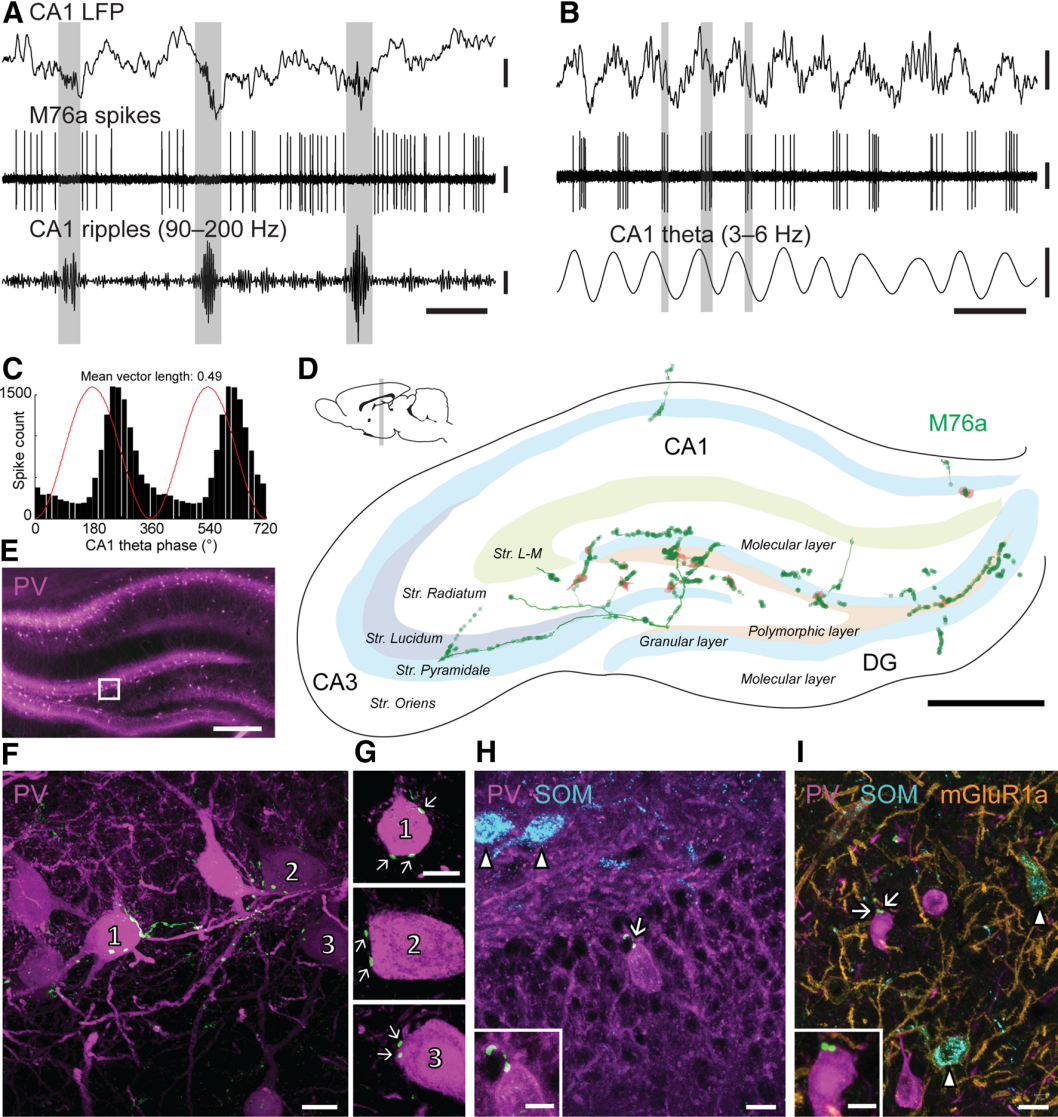
A SWR-suppressed medial septal neuron (M76a) mainly targeting PV-immunoreactive interneurons of the dorsal DG. **a** The neuron does not fire during SWR events (highlighted) recorded in the CA1. **b** The neuron fired preferentially along the descending phase of the CA1 theta cycles (some highlighted). **c** Theta phase histogram for spikes during all theta epochs (digitally duplicated; red line, sinus wave). **d** Reconstruction of part of the axon in the dorsal hippocampus four consecutive 70-µm-thick coronal sections. The axon (green) and terminal boutons (dots) of M76a are shown with putative interneuronal target somata (orange). **e** PV immunoreactivity in the hippocampal area. Framed region is sampled for the postsynaptic targets and enlarged in (**f**). **f** Three targeted PV-immunopositive somata (1, 2, 3) are located at the border of the dorsal granular and polymorphic layers of the DG. **g** Target somata and terminals (green/white dots, arrows) are shown in single optical sections or Z-stacks. Image heights: target 1 (top), single section of 0.32 µm; target 2 (middle), 0.65 µm; target 3 (bottom), 0.97 µm. **h, i** PV-positive somata in apposition to terminal boutons (arrows; enlarged in the insets) in the ventral granular layer (**h**) and in the stratum pyramidale of the anterior CA3 (**i**); nearby SOM-positive/PV-negative neurons (blue, arrowheads) were not contacted. Inset scale bars, 5 µm. **f, h, i** Maximum intensity projections of confocal image stacks; **f** 45.15 µm; **h** 9.97 µm; **i** 7.21 µm. Median filter was applied (x, y, z: radius 1 pixel) in F–I. *Str. L-M*, stratum lacunosum-moleculare; DG, the dentate gyrus. Vertical scale bars, 0.5 mV; except for band-pass filtered CA1 ripples at the bottom of A, 0.1 mV. Horizontal scale bars, 200 ms in A, 400 ms in B, 600 µm in D, 200 µm in E, 10 µm in F, H, I, 10 µm in G, insets of H and I

Testing possible preferential interneuron innervation outside CA1, we analyzed MS neuron M76a (Table 3) innervating the dentate gyrus (DG). Overall firing frequency was 22.31 Hz; it fired during only 31% of SWRs (*n* = 35; spikes/SWR = 0.38 ± 0.65, Fig. 4a). The overall firing rate during SWRs (4.48 ± 6.86 Hz) was much lower than that of “surrogate SWR” periods (24.13 ± 3.44 Hz; *p* < 0.0001, KS test; SWR index: − 0.69) suggesting inhibition. During the theta network state, this ‘septo-dentate’ neuron fired rhythmically (*r* = 0.49), phase-coupled to the descending phase of theta cycles (Fig. 4b, c; Table 4).

The axon innervated the septal pole of the DG and the adjacent CA3 (Scharfman 1995) with few collaterals innervating CA1 (Fig. 4d). Numerous small branches bearing varicosities were distributed across the molecular, granular and polymorphic layers of the DG (Fig. 4d). A sample of putative target interneurons were in the DG (*n* = 12), CA3 (*n* = 11) and CA1 (*n* = 2). Of the tested somata, 12/13 were immunopositive for PV (7, DG; 3, CA3; 2, CA1). None of the six PV-immunoreactive neurons (3, DG; 2, CA1; 1, CA3) tested for SATB1 were immunopositive. As SATB1 is expressed by BCs and bistratified cells but not AACs in CA1 and CA3 (Viney et al. 2013), the results suggest that PV+ targets are AACs. However, in the DG SATB1 is detectable in very few PV+ interneurons and is not a reliable differentiation marker for BCs and AACs. Therefore, we tested other PV-positive target neurons (2, DG; 1, CA3) for SOM, a marker for bistratified cells, and did not detect any immunoreactivity. We also tested 3 target neurons for immunoreactivity to CCK, 1 neuron for CR and 3 other target profiles for nNOS, and with one exception (a single nNOS-positive target profile in the polymorphic layer of DG) they were immunonegative. Consequently, in all three innervated areas, this septo-dentate neuron showed preferential targeting of PV-immunopositive interneurons. The border of the dorsal granular and polymorphic layer of the DG (Fig. 4e) contained three such PV-positive target neurons (Fig. 4f). Each of these target somata were contacted by at least two varicosities (Fig. 4g). Figure 4 also depicts a PV-immunoreactive and SOM-immunonegative target soma in the ventral granular layer (Panel H), and a PV-immunoreactive and SOM/mGluR1a-immunonegative target soma in stratum pyramidale of the anterior CA3 (Panel I).

### Dual innervation of the medial septum and CA3 by a SWR-active medial septal neuron (M82f)

**Fig. 5:**
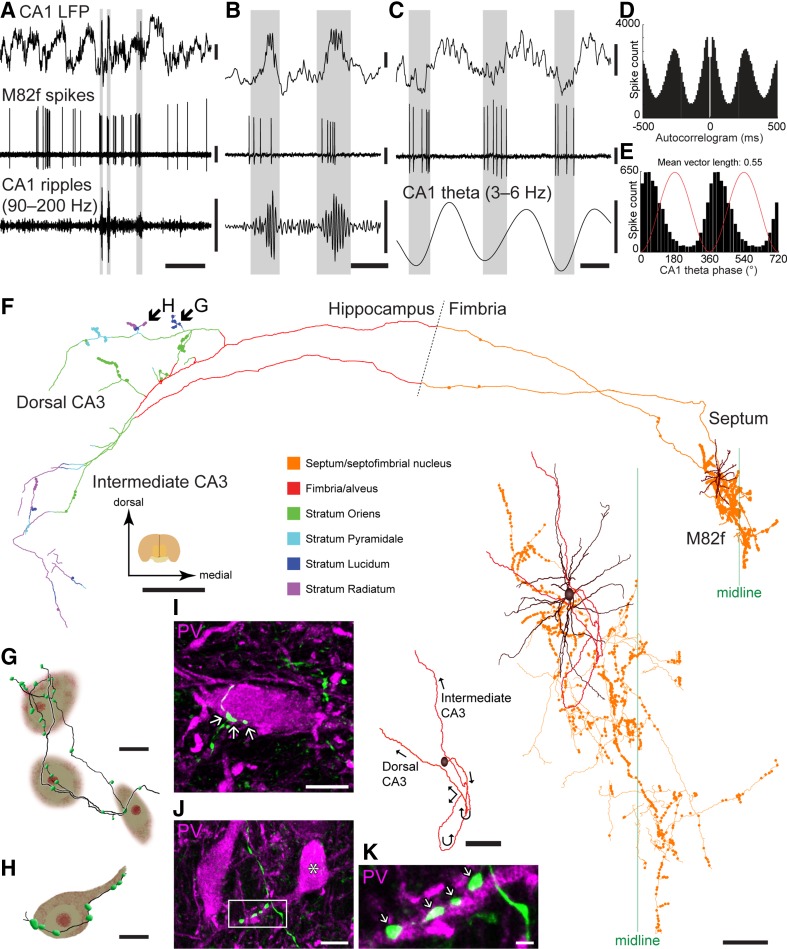
A SWR-active GABAergic neuron (M82f) with a dense local axonal arborization in the MS and projection to the CA3. **a, b** The neuron fires in bursts during SWR events in the CA1 LFP (highlighted) shown at two time scales. **c** During theta oscillations, the neuron fires in bursts (highlighted) phase-locked to the trough/early-ascending phase of the CA1 theta cycles. **d** Autocorrelogram of M82f during theta epochs showing strong theta modulation of firing. **e** Histogram showing preferential firing at the trough/early-ascending phase for detected theta cycles (shown duplicated across two cycles). **f** Digital reconstruction of the soma and dendrites (brown) in the MS. The axon was partially labeled and the varicosities (circles) are color-coded by area/hippocampal stratum as shown in the legend. Enlarged image shows that the soma and dendrites are located on the left side together with the CA3-projecting main axon (red), while the local axonal branches and varicosities (orange) cover both sides of the MS. The main axon is shown separately illustrating its hooked profile and two main projecting branches. **g, h** Reconstructions of the small parts (arrows in **f**) of the axon (black) and varicosities (green) with putative target interneuron somata in stratum lucidum of dorsal CA3. **i–k** Terminals (arrows) in the MS in apposition to a PV-immunoreactive soma (**i**) and a proximal dendrite of another PV-immunoreactive neuron (asterisk, **j**). The boxed region is shown in **k** as a single optical section. Median filter was applied (*x, y*: radius 1 pixel) in **i**–**k. i, j** Maximum intensity z-projection of confocal image stacks; each 6.7 µm-thick. Vertical scale bars, 0.4 mV. Horizontal scale bars, 1 s in **a**, 100 ms in **b, c**, 500 µm in **f**, 100 µm in insets of **f**, 10 µm in **g**–**j**, 2 µm in **k**

Neuron M82f was immunoreactive for PV, SATB1 and HCN4 in addition to VGAT (Viney et al. 2013; Table 3), and activated during SWRs (23.42 ± 15.69 Hz; *n* = 27 SWRs, vs. 9.64 ± 2.81 Hz “surrogate SWRs”; *p* < 0.0001, KS test; SWR index: 0.42; Fig. 5a, b). It was modulated during theta oscillations (*r* = 0.55; Fig. 5d, e; Viney et al. 2013), preferentially fired at the early-ascending phase (Fig. 5c, e), and increased its firing rate compared to non-theta periods (from 10.69 to 23.05 Hz; Fig. 1g).

The neuron had a dense local axonal arborization in the MS (Fig. 5f). Likewise, eight of thirteen labeled MS cells had local axon collaterals in septal nuclei (Table 2). Local axonal branches gave rise to numerous varicosities, both en passant and terminal, around the midline of the MS (Fig. 5f). Axonal targets included somata (Fig. 5i) and dendrites of PV+ neurons (Fig. 5j, k). In CA3, the axonal field was restricted to the lateral part within the septal and intermediate parts of the hippocampus, where all layers except stratum lacunosum-moleculare were innervated (Fig. 5f). Most boutons were not apposed to somata, but we observed rare somatic targets in CA3 stratum lucidum and radiatum (Fig. 5g, h). Thus, this SWR-active ‘septo-CA3’ neuron acts in restricted regions of the CA3 as well as locally in the MS. Based on its preferential firing to the theta trough and its target regions, this neuron may be a homolog of mouse ‘Teevra cells’ (Joshi et al. 2017; Viney et al. 2013).

### Exclusive innervation of dorsal CA3 by a basket cell-targeting medial septal neuron (M44)

**Fig. 6:**
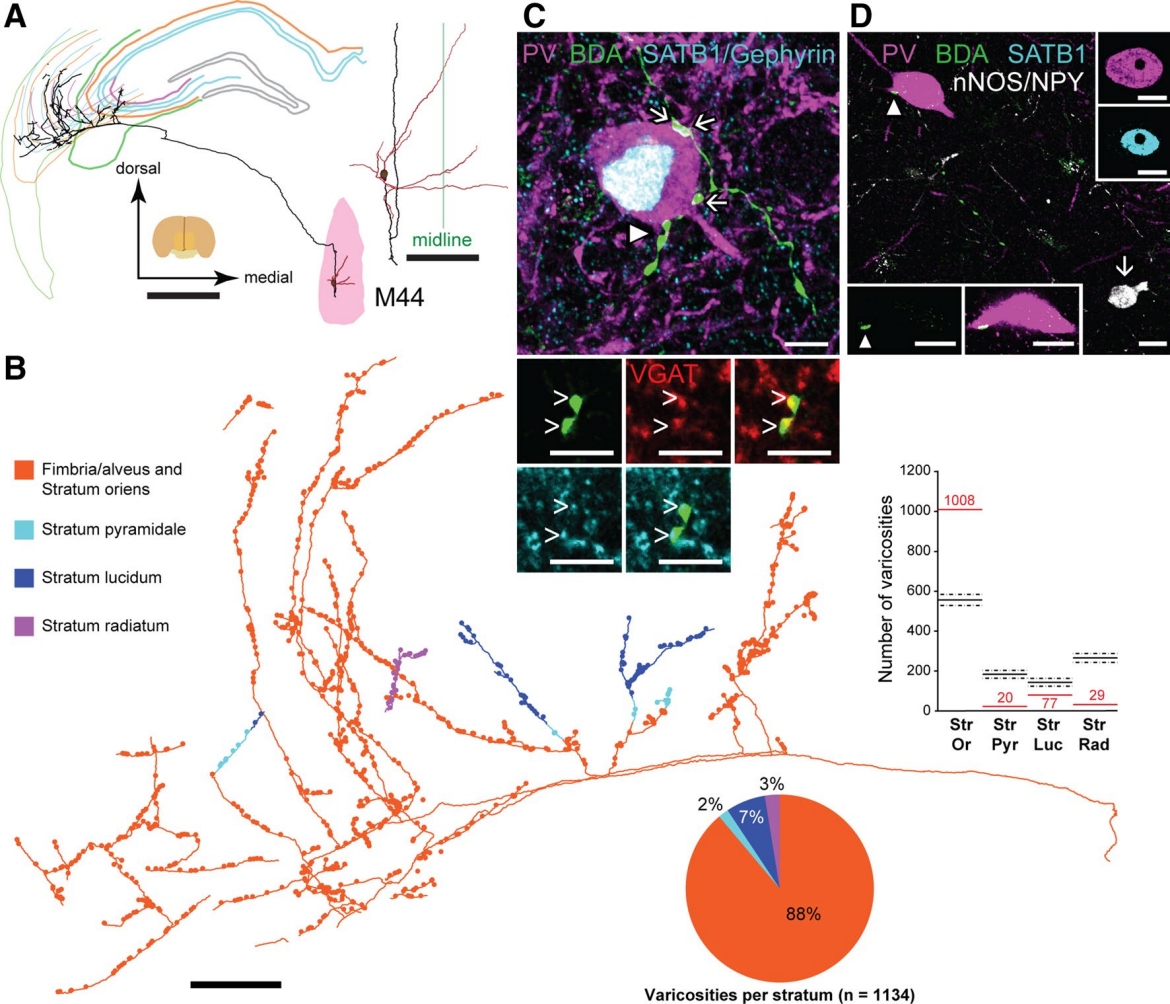
Hippocampal target area and postsynaptic target cell type selectivity of a medial septal GABAergic neuron (M44). **a** Digital reconstruction of the complete neuron shows the soma and dendrites (brown) in the MS (pink), and the axon (black) exclusively innervating exclusively dorsal CA3. Partial contours of the hippocampus are shown at different coronal levels; green lines, fimbria; orange, stratum oriens; blue, stratum pyramidale. **b** Axon and varicosities (dots) color-coded by hippocampal strata. The vast majority of boutons innervate stratum oriens and the alveus (pie chart). Bar plot: recorded laminar distribution of varicosities (red), and the simulated numbers expected (black; dashed lines show 95% confidence interval) under the assumption of uniformity between the different layer. Differences in the expected numbers are due to different volumes of innervated layers. **c** A BC immunoreactive for PV (purple) and SATB1 (cyan/white, nucleus) is innervated by septal boutons (green, arrows) in stratum pyramidale. Two boutons (single optical sections) labeled by BDA (green, arrowhead) are immunopositive for VGAT (red) and are adjacent to gephyrin-positive puncta (cyan). **d** Another PV/SATB1-immunoreactive BC in stratum oriens is innervated (arrowhead) by the axon. The BC is immunonegative for NPY and nNOS, which were tested sequentially in the same fluorescence channel. A nNOS-immunopositive soma in lower right (arrow) was not targeted. The inset at lower left shows the BDA positive bouton (arrowhead) in a single optical section. Inset at the top right side shows single confocal images of SATB1 immunoreactivity in the nucleus of the target neuron. **c, d** Maximum intensity projection of confocal image stacks; **c** 8.22 µm; **d** 12.07 µm. Median filter was applied (*x, y, z*: radius 1 pixel) in **c, d**. Scale bars, 1 mm in **a** 200 µm in the inset of, **a, b** 5 µm in **c**, 10 µm in **d**

A fully labeled GABAergic MS neuron (M44) showed remarkable target area and layer specificity. The neuron was immunopositive for PV, HCN4 and VGAT, and immunonegative for NECAB1. Some of its boutons were tested for VGluT2 and VAChT and lacked detectable immunoreactivity for both molecules (Table 3). The main axon projected through the fimbria branching in a restricted area of CA3b (Fig. 6), and although three sections could not be evaluated, a total of 1134 axonal varicosities were observed, mostly in the fimbria/alveus or stratum oriens (Fig. 6b). The distribution of varicosities by strata was not uniform (Fig. 6b, Chi square test, *χ*^2^ (3, *n* = 1134) = 762.137; *p* < 0.001).

We have analyzed 99 axonal varicosities in apposition to putative targets (Fig. 6c, d). Those tested formed GABAergic synapses, as shown by immunoreactivity for gephyrin (*n* = 45/45; Fig. 6c) and VGAT (*n* = 26/26; Fig. 6c). The majority of immunopositive targets were dendrites (*n* = 63), while fewer were somata (*n* = 22) and the targets of 14 boutons lacked a positive molecular marker. Combinations of molecular cell type markers revealed 12 PV+ and 1 PV− putative target neuron. In contrast to the CA3 targets of PV+ medial septal Teevra cells in mouse (Joshi et al. 2017), five of the PV-immunopositive neurons were also immunoreactive for SATB1 (Fig. 6c, d), but immunonegative for NPY (Fig. 6d) identifying them as BCs. Three other PV+ neurons not tested for SATB1 were immunonegative for SOM. Three additional PV+ neurons not tested for SATB1 or SOM, were immunonegative for mGluR1a, a marker for several interneuron types including some bistratified cells. These latter six neurons could be PV+ BCs. Only one PV+ neuron was immunopositive for SOM and mGluR1a, predicting a putative bistratified or O-LM cell. The only PV-immunonegative target contacted by a single varicosity was immunopositive for nNOS, hence a putative Ivy cell (Fuentealba et al. 2008). The above results show that in contrast to Teevra cells and the sept-CA3 neuron M82f above, this kind of septo-CA3 neuron has a strong preference for innervating PV+ BCs.

## Discussion

Cortical LFP oscillations reflect synchronization of neuronal activity across various timescales, which guide behavior through predictive temporal coding of events in the world (Hasselmo 2005; Buzsaki and Moser 2013; Fernandez-Ruiz et al. 2017; Kang et al. 2017). Theta frequency oscillations generated in the brainstem and midbrain are transferred to cortex through the basal forebrain including the MS (Vertes and Kocsis 1997; Kocsis and Kaminski 2006; Hangya et al. 200; Orzel-Gryglewska et al. 2015). The MS innervates cortical fields through cholinergic, GABAergic and glutamatergic projections (Gritti et al. 1997; Detari et al. 1999; Duque et al. 2000; Sotty et al. 2003; Henny and Jones 2008; Sun et al. 2014; Zaborszky et al. 2015), which are necessary to maintain behavioral performance and switching brain states (McNaughton et al. 2006; Roland et al. 2014; Kang et al. 2017). Previous population tracing studies revealed the target region selectivity and topographical organization of basal forebrain projections (Alonso and Kohler 1984; Zaborszky et al. 1999; Unal et al. 2015; Kondo and Zaborszky 2016). Septal glutamatergic (Huh et al. 2010; Fuhrmann et al. 2015) or GABAergic (Kaifosh et al. 2013) projections to unknown interneurons in CA1 and to pyramidal neurons in CA3 (Huh et al. 2010) modulate feed-forward inhibition and excitation of CA1 pyramidal cells. However, the intra- and inter-areal cortical termination of these pathways at single cell resolution are largely unknown. To our knowledge, only one study reported the axonal distribution of single basal forebrain cholinergic neurons in the hippocampal formation (Wu et al. 2014), and a recent study (Joshi et al. 2017) demonstrated the selective innervation of the hippocampal CA3 area by GABAergic medial septal Teevra cells in the mouse.

We have demonstrated that individual GABAergic neurons project to discreet regions of the hippocampus and show temporally specific activity patterns. Notwithstanding the technical challenge of labeling neurons to their termination zones, the successful examples revealed that (1) single MS GABAergic neurons terminate only in one or two areas, (2) single MS GABAergic neurons target specific GABAergic cell types, and (iii) the firing of single MS neurons, which differ during SWR oscillations also differ during the theta oscillatory state. These results provide some explanation for the coordination of network activity in the temporal lobe.

The MS neurons are highly diverse in their spike shapes, firing rates, and phase relationship to the hippocampal theta rhythm (Green and Arduini 1954; Petsche et al. 1962; Alonso et al. 1987; King et al. 1998; Borhegyi et al. 2004; Simon et al. 2006) and SWRs (Borhegyi et al. 2004; Viney et al. 2013). However, the MS innervates many cortical and subcortical areas and it remains to be determined which individual neurons project to the hippocampus and/or extrahippocampal brain areas. This requires the visualization of the axons of recorded single neurons (e.g., Joshi et al. 2017). Identified septal cholinergic neurons show low firing rates and long duration spikes, similar to the majority of basal forebrain cholinergic neurons (Detari et al. 1999; Manns et al. 2000b; Jones 2004; Unal et al. 2012). Glutamatergic neurons of the MS include slow-, fast-, cluster- and burst-firing neurons that can show spontaneous rhythmicity at theta frequencies (Huh et al. 2010; Justus et al. 2017). To reveal the projections of GABAergic neurons, we have selected highly rhythmic MS neurons, some of which were previously shown to be immunopositive for PV, which represents a subpopulation of GABAergic neurons (Morris et al. 1999; Henderson et al. 2004; Borhegyi et al. 2004), or VGAT (Manns et al. 2000a, 2003; Henny and Jones 2008). Indeed, by detecting VGAT immunoreactivity in the axon terminals of rhythmically firing neurons, we have conclusively demonstrated their GABAergic phenotype.

The hippocampal network generates temporally ordered neuronal firing during SWRs. Some hippocampal GABAergic neurons increase their firing rate during ripples and show phase coupling to the oscillatory cycles, while others are inhibited (Csicsvari et al. 1999; Klausberger and Somogyi 2008). Individual MS GABAergic neurons also show different firing rates during SWRs. The population of SWR-inhibited MS neurons was suggested to lead to increased firing of some hippocampal interneurons (Dragoi et al. 1999; Borhegyi et al. 2004), whereas MS neurons with increased SWR-related firing were proposed to inhibit AACs leading to disinhibition of pyramidal cells (Viney et al. 2013). Our sample of SWR-active and SWR-suppressed medial septal neurons represent distinct populations, since most of the latter were phase-coupled to the descending phase of CA1 theta oscillatory cycles, whereas the majority of former preferentially fired at the ascending phase. Furthermore, the SWR-suppressed group sustained its firing rate between theta and non-theta epochs, while SWR-active neurons showed increased activity during theta oscillations.

The diversity of MS GABAergic neurons may be explained by their projection to different cortical areas and/or different cell types within the same area, as demonstrated in the mouse hippocampus (Joshi et al. 2017). Our direct evidence shows that in the rat individual septo-hippocampal GABAergic neurons preferentially fire at different phases of CA1 theta oscillations and that these neurons implement theta rhythmic GABAergic influence through multiple parallel channels via target interneurons. We have confirmed the hypothesis of Borhegyi et al. (2004) that at least some of theta peak firing medial septal GABAergic neurons innervate GABAergic neurons in CA1, such as the bistratified cell, which selectively terminate on the dendrites of pyramidal cells (Halasy et al. 1996). This is likely to be a strong contribution to the specific theta phase-coupled firing of their target interneuron types (Klausberger and Somogyi 2008). We have based our prediction of synaptic junctions on the proximity of septo-hippocampal boutons and immunopositive somatic and/or dendritic profiles. Gephyrin immunoreactive patches in close association with GABAergic boutons, and at the interface of postsynaptic elements and boutons are highly reliable predictors of synaptic junctions (Viney et al. 2013; Panzanelli et al. 2011; Triller et al. 1985). However, as not all interfaces between immunohistochemically labeled putative postsynaptic dendrites and neurobiotin-labeled boutons were tested for gephyrin, some uncertainty remains about the presence of synapses between some close appositions, e.g., rarely encountered calretinin positive dendrites and neurobiotin-labeled septo-hippocampal boutons.

The pioneering study of Freund and Antal (1988) established that GABAergic neurons are the targets of the GABAergic septo-hippocampal projection (Kohler et al. 1984; Sun et al. 2014; Wu et al. 2014), and most interneuron types received such GABAergic input (Freund and Buzsaki 1996; Takács et al. 2008). However, the relationship of theta phase preference of the presynaptic septal neurons and the identity of their target interneurons has remained unknown due to technical challenges. Most interneurons in the hippocampus (Klausberger and Somogyi 2008) and entorhinal cortex (Quilichini et al. 2010) fire strongly phase-coupled to theta oscillations, but not all interneuron types have been recorded in vivo. Those types that were recorded had preferred theta firing phases, and the distinct types collectively covered the entire theta cycle with various depth of phase modulation (Csicsvari et al. 1999; Klausberger and Somogyi 2008). It has remained a puzzle if and how the theta rhythmic GABAergic input from the septum could contribute to the different theta phase entrainment of distinct interneurons. As individual theta rhythmic septal GABAergic neurons can have different theta phase preferences (King et al. 1998; Dragoi et al. 1999), it is possible that each septal neuron innervates only a restricted range of hippocampal interneuron types, and the theta phase preference of individual septal neurons and their postsynaptic targets are correlated (Alonso et al. 1987). Indeed, based on a roughly 180 degrees of difference in the theta phase preference of two populations of septal rhythmic neuron, the postsynaptic target cell type selectivity of septo-hippocampal neurons have been suggested (Borhegyi et al. 2004), but has remained untested. Our analysis provides the first evidence that a GABAergic septal neuron preferentially innervated PV+ neurons as compared to nNOS-expressing cells, and out of the 5 known PV-expressing interneuron types in CA1 (Klausberger and Somogyi 2008), it preferentially targeted bistratified cells.

Bistratified cells act through GABA_A_ receptors on dendrites in association with the glutamatergic CA3 input to CA1 pyramidal cells. We identified bistratified cells, which preferentially fire at the trough of theta cycles (Klausberger et al. 2004), as preferential targets of a presynaptic GABAergic septal neuron that preferred the peak of the theta cycle. This suggests that the septal GABAergic neuron inhibits its postsynaptic target interneurons (Freund and Antal 1988), and is likely to contribute to their theta firing phase restriction. The target interneuron preference was not restricted to the CA1 innervating septal GABAergic neuron. The synaptic targets of M44, which innervated a restricted area of CA3, were PV+ BCs. This is remarkable because another prominent PV+ interneuron in CA3, the AAC, is selectively innervated by GABAergic axons from the MS (Viney et al. 2013; Joshi et al. 2017). Thus, it is likely that PV+ CA3 BCs and AACs, which differ in their theta phase firing preference, receive input from separate GABAergic MS neurons.

Our demonstration of local axonal collaterals of GABAergic neurons and synaptic targeting of PV+ neurons in the MS is consistent with previous reports (Henderson et al. 2001; Borhegyi et al. 2004). The projection areas of postsynaptic PV+ neurons and their theta firing phases remain to be determined (Borhegyi et al. 2004). It is possible that the local axonal innervation in the MS contributes to synchronization of septo-hippocampal neurons firing on the same theta phase, and projecting to the same or different cortical areas. This would be analogous to the mechanism of synchronization of theta rhythmic PV+ BCs in the hippocampus, which innervate each other (Freund and Buzsaki 1996).

In conclusion, oscillatory neuronal activity, reflecting temporal windows of increased and decreased excitability, signifies inter-regional communication in the brain and is coupled to cognitive processes (Hasselmo 2005; Jones and Wilson 2005; Dragoi and Buzsaki 2006). For instance, theta rhythmically firing time cells of the hippocampus might represent the temporal dimension of episodic memories. Subcortical GABAergic afferents of the MS selectively innervating specific GABAergic interneurons provide a powerful link for the oscillatory synchronization of large populations of neurons (Fernandez-Ruiz et al. 2017). Revealing the axonal target areas and target cells demonstrated a diverse set of highly selective and specialized septal neurons contributing to different oscillatory brain states. The axons of the two fully labeled individual cells, like those reported in the mouse (Joshi et al. 2017), innervated restricted areas of the hippocampus. The other partially labeled axons also passed through large hippocampal areas without emitting collaterals or forming boutons. This suggests that septo-hippocampal GABAergic neurons act in a functional area restricted manner via select rhythmically active postsynaptic interneurons participating in the temporal structuring of the hippocampal network (Somogyi et al. 2014).
